# Central Sensitization and Perceived Indoor Climate among Workers with Chronic Upper-Limb Pain: Cross-Sectional Study

**DOI:** 10.1155/2015/793750

**Published:** 2015-09-06

**Authors:** Emil Sundstrup, Markus D. Jakobsen, Mikkel Brandt, Kenneth Jay, Roger Persson, Lars L. Andersen

**Affiliations:** ^1^National Research Centre for the Working Environment, 2100 Copenhagen, Denmark; ^2^Institute for Sports Science and Clinical Biomechanics, University of Southern Denmark, 5230 Odense, Denmark; ^3^Physical Activity and Human Performance Group, SMI, Department of Health Science and Technology, Aalborg University, 9220 Aalborg, Denmark; ^4^Department of Psychology, Lund University, Box 213, 221 00 Lund, Sweden

## Abstract

Monitoring of indoor climate is an essential part of occupational health and safety. While questionnaires are commonly used for surveillance, not all workers may perceive an identical indoor climate similarly. The aim of this study was to evaluate perceived indoor climate among workers with chronic pain compared with pain-free colleagues and to determine the influence of central sensitization on this perception. Eighty-two male slaughterhouse workers, 49 with upper-limb chronic pain and 33 pain-free controls, replied to a questionnaire with 13 items of indoor climate complaints. Pressure pain threshold (PPT) was measured in muscles of the arm, shoulder, and lower leg. Cross-sectional associations were determined using general linear models controlled for age, smoking, and job position. The number of indoor climate complaints was twice as high among workers with chronic pain compared with pain-free controls (1.8 [95% CI: 1.3–2.3] versus 0.9 [0.4–1.5], resp.). PPT of the nonpainful leg muscle was negatively associated with the number of complaints. Workers with chronic pain reported more indoor climate complaints than pain-free controls despite similar actual indoor climate. Previous studies that did not account for musculoskeletal pain in questionnaire assessment of indoor climate may be biased. Central sensitization likely explains the present findings.

## 1. Introduction

Previous research has documented the importance of a good indoor climate for workers health and safety [1–4]. Effective occupational health and safety programs include workplace assessments, for example, monitoring the indoor climate by asking the workers about complaints such as noise, draught, and temperature. Such analyses can identify physical hazards related to job position or work location and thereby provide key knowledge to preventing work-related health hazards [3]. However, workers with chronic pain may experience the indoor climate differently than pain-free colleagues which could distort conclusions. Thus, to avoid any misinterpretation of workplace health and safety programs and to prevent further worsening of symptoms it is important to identify workers with high prevalence of indoor climate complaints along with the specific bothering factors. Previous studies on indoor climate have mainly focused on symptoms attributed to indoor environment by comparing different workplaces or job positions [3, 5–7]. However, knowledge concerning interindividual variation in indoor climate sensation as a consequence of chronic musculoskeletal pain is lacking.

Chronic musculoskeletal pain is a common problem that affects millions of people worldwide. The consequences of chronic pain are often serious, affecting employee health and wellbeing, and impose a substantial socioeconomic burden due to extensive use of health care services, sickness absence, disability pension, and loss of productivity [8, 9]. Initially pain may emanate from activation of peripheral nociceptors due to tissue damage, but when the perception of pain for some reason (e.g., untreated pain, poor pain treatment, or just too long activation of the pain system) persists beyond the expected time for tissue healing, it has become chronic [10]. Chronic pain is caused by not only physiological pathology but rather a complex interaction between biological, psychological, and social factors [10, 11]. This implies the existence of many workplace risk factors for chronic pain and emphasizes the necessity of an effective and inclusive occupational health and safety system to rehabilitate and prevent aggravation of symptoms. Central sensitization is an important mechanism involved in chronic pain conditions and is defined as “facilitated excitatory synaptic response and depressed inhibition, causing amplified responses to noxious and innocuous inputs” [12, 13]. Thus, central sensitization may not only influence perception of pain, but also amplify other nonpainful stimuli. In line with this, de Klaver and colleagues [14] demonstrated that hyperacusis (intolerance of ordinary sound levels) is common among patients severely affected by complex regional pain syndrome type 1. Hyperacusis has been associated with central nervous system involvement and the authors speculated that hyperacusis may reflect the spreading of central sensitization to auditory circuits in these patients. We have also observed central sensitization in women with frequent neck-shoulder pain and desensitization in response to physical rehabilitation [15]. Also patients with chronic pain conditions such as fibromyalgia and irritable bowel syndrome have been shown more responsive to other stimuli than pain (auditory, visual) suggesting that these individuals have a different interpretation of pain or sensory information that reaches further than to the specific body region where the pain is perceived to be situated [16]. Thus, it could be speculated that central sensitization may spread to sensory processing circuits in workers with chronic pain consequently affecting their perception of specific indoor climate factors.

Slaughtering and meat processing operations involve a high degree of repetitive and forceful upper-limb movements and imply an elevated risk of work-related musculoskeletal disorders [17, 18]. In particular the prevalence of musculoskeletal disorders in the shoulder, arm, and hand is high among slaughterhouse workers, allegedly due to frequently repeated high-force actions and lack of sufficient recovery of these body regions during work [19–23]. Additionally, to comply with the standards on meat processing, ambient temperature is often low increasing the risk of MSD. Previous studies have demonstrated that musculoskeletal complaints, such as shoulder pain and low back pain, are more prevalent when the work is performed in a cold environment compared with work performed in normal temperature and that these symptoms seem to be aggravated as a function of time working in cold conditions [24–26]. Specifically, studies among seafood industry workers have shown that a cold work environment is a risk factor for musculoskeletal symptoms [27] and that workers who often felt cold at work experienced a higher prevalence of musculoskeletal pain compared to workers who never felt cold [6]. The authors concluded that the prevalence of feeling cold at work could be a valuable estimate for exposure in moderate cold exposure situations [6]. In addition to low working temperature, other environmental risk factors for MSD at the slaughterhouse involve draught from compressing machinery or elsewhere as well as noise from the mechanically based production systems. It is, however, unknown whether slaughterhouse workers with chronic pain are more sensitive to these environmental risk factors and to what extent central mechanisms are involved in the perception of these factors.

The aim of this study was to investigate whether workers with chronic pain compared with pain-free colleagues perceive the same indoor climate differently. We hypothesized that workers with chronic pain were more sensitive to the indoor climate (especially cold, draught, noise, and light) and that central sensitization could be involved in this perception.

## 2. Materials and Methods

### 2.1. Study Design and Ethics

A cross-sectional study concerning central mechanisms to chronic pain and its relation to perceived indoor climate complaints was conducted among 82 male slaughterhouse workers in Denmark, Europe. The present study was a part of a randomized controlled trial on the effect of strength training or ergonomic counseling on chronic pain [23] and was approved by the Danish National Ethics Committee on Biomedical Research (Ethical Committee of Frederiksberg and Copenhagen, H-3-2010-062) and registered in ClinicalTrials.gov (NCT01716767). All participants were informed about the purpose and content of the project and gave their written informed consent to participate in the study. All experimental conditions conformed to the Declaration of Helsinki.

### 2.2. Participants

Eighty-two male slaughterhouse workers, 49 with chronic upper-limb pain and 33 pain-free controls, with similar job tasks were recruited from two large scale pig slaughterhouses for the study. Participant characteristics for the two groups are shown in Table 1. The workers were recruited in relation to the before mentioned intervention study [23, 28].

The recruitment process was two-phased and consisted of a brief screening questionnaire followed by a clinical examination and questionnaire. The screening questionnaire was administered to 645 Danish slaughterhouse workers and contained questions on demographics, pain, and diagnosed diseases. Those who were interested in participating and who met the initial inclusion criteria for one of the two groups (chronic pain or pain-free controls, criteria described later) were invited for the clinical examination and questionnaire. Table 1 shows demographics, clinical, and work-related characteristics of the participants in the two groups.

Participants in the chronic pain group were to display (1) pain intensity in the shoulder, elbow/forearm, or hand/wrist of 3 or more on a 0–10 VAS scale during the last 3 months, (2) pain intensity in the shoulder, elbow/forearm, or hand/wrist of 3 or more on a 0–10 VAS scale during the last week, (3) pain that has lasted more than 3 months, (4) frequency of pain of at least 3 days per week during the last week, and (5) statement of at least “*some*” work disability scoring on a five-point scale, “*not at all*,” “*a little*,” “*some*,” and “*much*” to “*very much*,” when asked the question “*During the last 3 months, did you have any difficulty performing your work due to pain in the shoulder, arm or hand?*” This single-item question was inspired by the work module of the DASH questionnaire [29].

The pain-free controls were a comparison group showing no pain symptoms or work disability. Inclusion criteria for this group were as follows: (1) pain intensity in the shoulder, elbow/forearm, or hand/wrist of 1 or less during the last 3 months, (2) pain intensity in the shoulder, elbow/forearm, or hand/wrist of 1 or less during the last week, and (3) statement of “*not at all*” work disability scoring on a five-point scale, “*not at all*,” “*a little*,” “*some*,” and “*much*” to “*very much*,” when asked the question “*During the last 3 months, did you have any difficulty performing your work due to pain in the shoulder, arm or hand?*”

Exclusion criteria for all participants were hypertension (systolic BP > 160, diastolic BP > 100), a medical history of cardiovascular diseases, symptoms of carpal tunnel syndrome, recent traumatic injury of the neck, shoulder, arm, or hand regions, or pregnancy.

### 2.3. Experimental Procedure

After the initial screening questionnaire, eligible participants were asked to present at the slaughterhouse for a questionnaire and clinical examination. In the questionnaire, participants were to rate the prevalence of indoor environmental complaints by the MM-indoor climate questionnaire [30, 31]. The MM-questionnaire contained questions on 13 different indoor environmental factors, and the participant had to rate whether the single factor is a bothering workplace-factor by stating “yes, often,” “yes, sometimes,” or “no, never.” Only the alternative “yes, often” (meaning every week) was used as a positive outcome in the analyses. The 13 indoor environmental factors were draught, room temperature too high, varying room temperature, room temperature too low, stuffy “bad” air, dry air, unpleasant odour, static electricity, passive smoking, noise, lighting problems, reflections, and dust and dirt. Additionally, participants were asked about year of birth, height and weight, seniority, and primary job function. Primary job function was further trichotomized into “meat cutters,” “meat packers,” and “slaughterer.” Smoking habits were identified by the question “do you smoke?”—“yes” or “no.” Further, work disability was rated by the work module of the Disability of the Arm, Shoulder and Hand (DASH) questionnaire [23, 29].

The clinical examination of participants included pressure algometry using an electronic pressure algometer (Somedic Productions AB, Sollentuna, Sweden, Europe). The examiner was blinded to group allocation and measured pressure pain threshold (PPT) of the painful muscles (infraspinatus and extensor carpi radialis brevis) and a nonpainful reference muscle (tibialis anterior). The contact area of the circular probe was 1 cm^2^ and pressure was applied perpendicular to the skin at the mid-belly of the 3 muscles at a rate of 30 kPa·s-1 [15]. The participant was not aware of the reading of PPT on the display and was instructed to push the patient operated switch on a pinch handle mounted on the algometer when the sensation of “pressure” changed to “pain”. PPT was measured 3 times at the infraspinatus, extensor carpi radialis brevis, and tibialis anterior with 1.5 min between each measurement alternating between the 3 muscles [15]. PPT for each muscle was subsequently expressed as the average value of the 3 measurements. Previous studies have shown satisfactory to good test-retest reliability of PPT [32, 33].

### 2.4. Statistical Analysis

Pearson chi-square tests were performed to investigate between-group differences in indoor climate complaints for each of the 13 environmental factors by the use of the frequency procedure of SAS version 9.3.

Using general linear models (PROC GLM of SAS version 9.3) we estimated the relationships between indoor climate complaints, PPT, and chronic pain in two sets of analyses. The first set of analyses included average number of indoor climate complaints as a dependent variable and job position (meat cutter, meat packer, and slaughterer) and smoking habits (yes, no) as independent classification variables. In model 1, the relationship between indoor climate complaints and chronic pain was investigated by including group allocation (chronic pain, pain-free controls) as an independent classification variable. In model 2, the relationship between indoor climate complaints and central sensitization was investigated by adding tibialis anterior PPT (very low, low, moderate, and high) as an independent classification variable. Both models were adjusted for age.

The second set of analyses included PPT as a dependent variable and job position and smoking habits as independent classification variables. The analyses were adjusted for age. In model 3, we investigated the association between central sensitization and chronic pain by including PPT of tibialis anterior (pain-free muscle) as the dependent variable. In models 4 and 5, the relationship between peripheral sensitization and chronic pain was estimated by adding extensor carpi radialis brevis and infraspinatus (painful muscles) as dependent variables, respectively.

Results are given as least square means and 95% confidence intervals. An alpha level of less than 0.05 was accepted as statistically significant.

## 3. Results

Table 1 shows descriptive statistics for the main study variables. No difference existed in job position between participants in the two groups.

Table 2 summarizes specific indoor climate complaints for workers with chronic pain and the pain-free controls. A significantly greater proportion of workers with chronic pain reported higher prevalence of (“often”) draught (*p* < 0.001) and noise (*p* < 0.05) during work compared with the healthy controls (Table 2). There were no differences between the two groups for the remainder of the indoor climate factors.

Table 3 summarizes the relationship between average indoor climate complaints and chronic pain (model 1) and PPT of tibialis anterior (model 2). Model 1 explained 9% of the variance observed for average indoor complaints and shows that slaughterhouse workers with chronic pain reported twice as many indoor climate complaints as pain-free controls (95% CI 1.8 versus 0.9, *p* = 0.0163) corresponding to a between-group difference of 0.9 complaints (95% CI 0.2 to 1.5). Model 2 explained 14% of the variance observed for average indoor complaints and indicates a significant negative association between tibialis PPT and indoor climate complaints (*p* = 0.019). However, only “very low” PPT scores were associated with more complaints. Compared with “low to high” PPT workers, workers with “very low” tibialis PPT reported a twofold increase in indoor complaints (2.4 versus 1.2). We found no significant influence of job function, age, or smoking habits on indoor climate complaints for both models.

**Figure 1:**
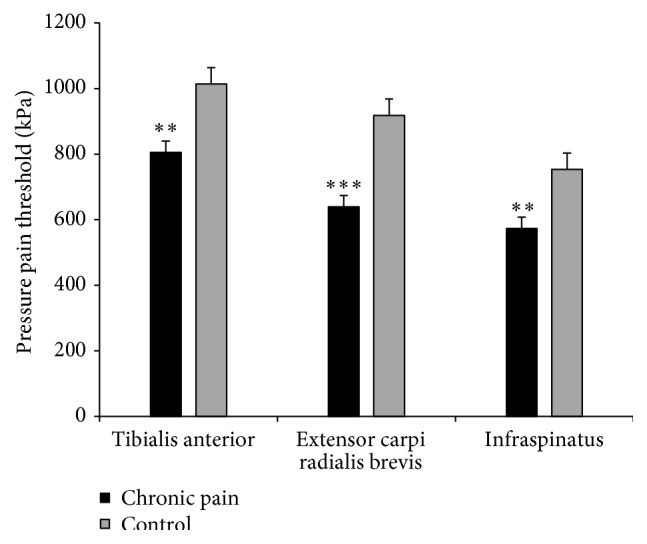
Average pressure pain threshold (PPT) of nonpainful (tibialis anterior) and painful (extensor carpi radialis brevis and infraspinatus) muscles among slaughterhouse workers with chronic pain and pain-free controls. *∗∗*,*∗∗∗* denotes difference from the pain-free control group (*p* < 0.01; *p* < 0.0001, resp.).

Table 4 summarizes the relationship between PPT of different muscles and chronic pain. PPT for both tibialis anterior and extensor carpi radialis brevis and infraspinatus were significantly lower in workers with chronic pain compared with pain-free controls (Figure 1). A significant association was observed for chronic pain and PPT of tibialis anterior, extensor carpi radialis brevis, and infraspinatus. The model explained 20%, 31%, and 23% of the variance observed for tibialis anterior PPT (model 3), extensor carpi radialis PPT (model 4), and infraspinatus PPT (model 5), respectively. We found no significant influence of job function, age, or smoking habits on PPT of any of the muscles tested.

## 4. Discussion

Workers with chronic pain reported twice as many indoor climate complaints as pain-free controls despite similar actual indoor climate. The number of complaints was associated with PPT of the nonpainful tibialis anterior suggesting a spreading of central sensitization to sensory processing circuits and thereby an amplification of external stimuli.

Workers with chronic pain displayed increased sensitivity to pressure, evidenced by lower PPT, in the painful muscles of the arm and shoulder compared with pain-free controls. This hyperalgesia may partly be explained by local cellular muscle mechanisms. However, in any chronic pain condition there is a fine tuned interplay of peripheral and central factors that can mutually influence and reinforce each other [16]. In line with this, we observed lower PPT in the nonpainful tibialis anterior among the workers with chronic pain reflecting a certain degree of general sensitization and thus the involvement of a central mechanism in the perception of upper-limb chronic pain. Generalized hypersensitivity, evidenced by enhanced nociceptive sensitivity and reduced pressure pain threshold, has also been reported in patients with chronic pain conditions such as fibromyalgia, trapezius myalgia, and chronic low back pain [15, 34, 35]. The proposed mechanisms of central sensitization involve an enhancement in the function of neurons and circuits in nociceptive pathways caused by increases in membrane excitability and synaptic efficacy as well as reduced inhibition [36].

The neural changes that lead to central sensitization may not only influence the perception of chronic pain, but also amplify other nonpainful stimuli [13, 36]. Hence, patients with chronic pain conditions have been shown more sensitive to other stimuli than pain, such as visual and auditory sensations [16]. This shows that these patients have a fundamental problem with pain or sensory processing rather than an abnormality confined to the specific body region where the pain is perceived to be situated [16]. In the present study, workers with chronic pain reported twice as many indoor climate complaints as healthy controls despite similar environmental working conditions. This higher complaint rate was additionally associated with lower PPT in the tibialis anterior suggesting that central sensitization may amplify normal innocuous factors to make them intolerable to workers with chronic upper-limb pain. Of the 13 single indoor climate factors of the indoor climate questionnaire, the chronic pain group reported significantly higher prevalence of bothering from draught and noise. Possibly this reflects a spreading of central sensitization to sensory processing circuits involved in noise and draught perception in workers with chronic pain. The existence of a biological amplification of sensory stimuli to chronic pain has support from functional imaging studies in patients with fibromyalgia that shows hyperactivity in the insula which plays a critical role in sensory integration and emotional processing of sensation [16, 37, 38]. Additionally, de Klaver and colleagues [14] demonstrated that hyperacusis (intolerance of ordinary sound levels) is common among patients severely affected by complex regional pain syndrome type 1. Hyperacusis has been associated with central nervous system involvement and the authors speculated that hyperacusis may reflect the spreading of central sensitization to auditory circuits in these patients.

To foster a safe and healthy work environment, occupational health and safety programs have strong focus on primary prevention of workplace hazards along with monitoring of workers symptoms and perception of the work environment. Altogether, these analyses can indicate the best mode of prevention and rehabilitate work-related illness and discomfort. Environmental hazards in the slaughterhouse involve, among others, low ambient temperatures, draught from compressing machinery or elsewhere, and noise from the mechanically based production systems. In the present study, the prevalence of complaints from draught and noise was higher among workers with chronic pain compared with pain-free controls, whereas no difference was observed for low temperature or light complaints. In contrast, Bang et al. [6] reported a large interindividual variation in cold sensation among seafood industrial workers in Norway and found that workers who often felt cold reported increased prevalence of musculoskeletal pain. However, no physiological measures were obtained to propose a mechanistic explanation and the authors speculated that the interindividual variation in cold sensation was due to variations in activity levels, clothing, and cold adaptation. Spreading of central sensitization to sensory circuits as a function of chronic pain could possibly explain the increased sensitivity to draught and noise as a function of chronic pain in the present study. However, from this perspective one would perhaps expect more complaints in relation to lighting and temperature. Surprisingly, however, central sensitization did not seem to influence the processing of cold or light sensation among the workers with chronic pain.

The observed existence of an interindividual variation in indoor climate sensation among workers with and without chronic pain suggests that conclusions of occupational health and safety programs that assess the indoor environment with self-reports may be distorted. Additionally, we found that persons with very low PPT, regardless of chronic pain, likewise experienced the indoor climate more bothering than subjects with higher PPT. Occupational physicians monitoring workers' symptoms and their perception of the work environment should take into account musculoskeletal pain and pressure soreness of employees in the evaluation of environmental workplace hazards. Additionally, future studies on indoor climate perception should be adjusted for musculoskeletal pain or pressure threshold.

## 5. Strength and Limitations

Our study has both strengths and limitations. Combining direct measures of pressure pain threshold with subjective reporting on indoor climate among workers with chronic pain is a strength. To our knowledge this is the first study to focus on the interindividual variance in indoor climate complaints as a function of chronic disease and additionally to offer a mechanistic explanation for this variance. A limitation is that we did not collect any objective data of the specific indoor climate factors such as draught and noise to support the workers perception of the indoor work environment. Because this part of the study was exploratory in nature, that is, assessing several indoor climate complaints, there is a chance of random findings. However, the *p* value of <0.001 for “draught” shows that even correcting conservatively for type I errors, for example, by Bonferroni correction, would not change the overall results. Furthermore, the complaints that reached significance are in agreement with those expected in the hypothesis. Unfortunately we did not have brain imaging to support the spreading of central sensitization to sensory processing circuits. However it would be optimal if future studies on chronic disease and climate perception could include brain imaging techniques to investigate whether hyperactivity exists in the areas of the brain involved in sensory processing. Even though the nociceptive responses to different types of painful stimuli largely converge, using only the tibialis anterior as reference could be a limitation. Finally, the cross-sectional design of the present study makes it impossible to conclude any causal relationship between the number of indoor climate complaints and central sensitization, and the generalizability of the results is limited to workers with heavy manual job tasks. Thus studying the longitudinal effect of climate complaints and pain with an additional focus on development of central sensitization would be of interest. It would also be relevant to investigate whether changes in pain over time follow changes in the perception of indoor climate among workers remaining in the same workplace with the same actual indoor climate.

## 6. Conclusion

Workers with chronic pain reported more indoor climate complaints than pain-free controls despite similar actual indoor climate. Previous studies that did not account for musculoskeletal pain in questionnaire assessment of indoor climate may be biased. Central sensitization likely explains the present findings.

## Figures and Tables

**Table 1:** Descriptive statistics for the main study variables. Mean (SD).

	Chronic pain (*n* = 49)	Control (*n* = 33)
Demographics		
Age (years)	45 (11)	45 (11)
Height (cm)	179 (7)	178 (7)
Weight (kg)	90 (16)	87 (13)
Body mass index (kg·m^2^)	28 (5)	28 (5)
Proportion of smokers (%)	41	25
Clinical		
Shoulder pain intensity in the previous week (0–10)	5.6 (2.3)	0.1 (0.3)
Elbow/forearm pain intensity in the previous week (0–10)	3.9 (2.7)	0.0 (0.2)
Hand/wrist pain intensity in the previous week (0–10)	3.7 (2.9)	0.2 (0.6)
Pain duration >3 months (%)	100	0
PPT tibialis anterior (kPa)	805 (205)	1014 (285)
PPT extensor carpi radialis brevis (kPa)	639 (147)	918 (201)
PPT infraspinatus (kPa)	573 (138)	753 (178)
Hand grip strength (Kg)	39 (14)	51 (10)
Work-related		
Weekly working hours	40 (1)	39 (6)
Duration of slaughterhouse work (years)	17 (10)	16 (13)
DASH work module (0–100)	28 (17)	0 (0)

**Table 2:** Prevalence of indoor climate complaints among slaughterhouse workers with chronic pain and pain-free controls.

	Chronic pain (*n* = 49)	Control (*n* = 33)
	Prevalence (%)	Prevalence (%)
Draught	40^*∗*^	6
Room temperature too high	2	3
Varying room temperature	4	3
Room temperature too low	24	15
Stuffy “bad” air	0	3
Dry air	6	6
Unpleasant odour	9	3
Static electricity	0	3
Passive smoking	4	0
Noise	58^*∗*^	33
Lightning problems	6	0
Reflections	6	6
Dust and dirt	9	9

Average complaints (mean; 95% CI)	1.8 (1.3–2.3)^*∗*^	0.9 (0.4–1.5)

^*∗*^Significantly different from the pain-free control group (*p* < 0.05).

**Table 3:** The relationship between average indoor climate complaints (dependent variable) and chronic pain (model 1) and pressure pain threshold (PPT) of tibialis anterior (model 2). We found no significant influence of job function, age, or smoking habits on indoor climate complaints for both models.

Dependent variable: number of indoor climate symptoms						
	Model 1			Model 2		
	LS mean	95% CI	*p* value (*F*-value)	LS mean	95% CI	*p* value (*F*-value)
Status			0.0163^*∗*^ (6.05)			
Chronic pain	1.8	1.3–2.3				
Pain-free	0.9	0.4–1.5				
Job position			0.5567 (0.59)			0.9613 (0.04)
Meat cutter	1.3	0.9–1.7		1.4	1.0–1.8	
Meat packer	1.1	0.4–1.7		1.4	0.7–2.0	
Slaughterer	1.6	0.7–2.7		1.5	0.5–2.6	
Smoking			0.9275 (0.01)			0.6404 (0.22)
Yes	1.3	0.7–2.0		1.5	0.9–2.2	
No	1.4	0.9–1.8		1.4	0.9–1.8	
PPT tibialis anterior						0.0190^*∗*^ (3.54)
Very low				2.4	1.6–3.1	
Low				0.9	0.1–1.7	
Moderate				1.2	0.5–1.9	
High				1.3	0.7–2.0	

^*∗*^Significantly associated to average indoor climate complaints (*p* < 0.05).

**Table 4:** The relationship between pressure pain threshold (PPT) of the different muscles and chronic pain. We found no significant influence of job function, age, or smoking habits on PPT for either of the muscles.

Dependent variable: PPT									
	Model 3			Model 4			Model 5		
	Tibialis anterior			Extensor carpi radialis brevis			Infraspinatus		
	LS mean	95% CI	*p* value (*F*-value)	LS mean	95% CI	*p* value (*F*-value)	LS mean	95% CI	*p* value (*F*-value)
Status			0.0023^*∗*^ (9.99)			<0.0001^*∗*^ (26.42)			0.0006^*∗*^ (13.03)
Chronic pain	805	701–910		639	554–725		573	494–652	
Pain-free	1015	900–1130		918	824–1013		753	666–839	
Job position			0.1021 (2.35)			0.1500 (1.95)			0.7458 (0.29)
Meat cutter	806	721–891		763	694–833		635	571–699	
Meat packer	877	751–1003		694	591–797		655	561–750	
Slaughterer	1047	841–1253		879	710–1048		698	543–854	
Smoking			0.0593 (3.67)			0.1830 (1.81)			0.0980 (2.81)
Yes	977	847–1107		817	711–924		707	609–805	
No	843	751–934		740	665–815		618	550–687	

^*∗*^Statistically significant association between the PPT of respective muscle and status (chronic pain versus pain-free control) (*p* < 0.05).
